# The AS87_04050 Gene Is Involved in Bacterial Lipopolysaccharide Biosynthesis and Pathogenicity of *Riemerella anatipestifer*

**DOI:** 10.1371/journal.pone.0109962

**Published:** 2014-10-10

**Authors:** Xiaolan Wang, Chan Ding, Shaohui Wang, Xiangan Han, Wanwan Hou, Jiaping Yue, Jiechi Zou, Shengqing Yu

**Affiliations:** Shanghai Veterinary Research Institute, Chinese Academy of Agricultural Sciences, Shanghai, China; Universidad Nacional de La Plata, Argentina

## Abstract

*Riemerella anatipestifer* is reported worldwide as a cause of septicemic and exudative diseases of domestic ducks. In this study, we identified a mutant strain RA2640 by Tn4351 transposon mutagenesis, in which the AS87_04050 gene was inactivated by insertion of the transposon. Southern blot analysis indicated that only one insertion was found in the genome of the mutant strain RA2640. SDS-PAGE followed by silver staining showed that the lipopolysaccharide (LPS) pattern of mutant strain RA2640 was different from its wild-type strain Yb2, suggesting the LPS was defected. In addition, the phenotype of the mutant strain RA2640 was changed to rough-type, evident by altered colony morphology, autoaggregation ability and crystal violet staining characteristics. Bacterial LPS is a key factor in virulence as well as in both innate and acquired host responses to infection. The rough-type mutant strain RA2640 showed higher sensitivity to antibiotics, disinfectants and normal duck serum, and higher capability of adherence and invasion to Vero cells, compared to its wild-type strain Yb2. Moreover, the mutant strain RA2640 lost the agglutination ability of its wild-type strain Yb2 to *R. anatipestifer* serotype 2 positive sera, suggesting that the O-antigen is defected. Animal experiments indicated that the virulence of the mutant strain RA2640 was attenuated by more than 100,000-fold, compared to its wild-type strain Yb2. These results suggested that the AS87_04050 gene in *R. anatipestifer* is associated with the LPS biosynthesis and bacterial pathogenicity.

## Introduction

*Riemerella anatipestifer* is a Gram-negative, non-motile, nonspore forming, rod-shaped bacterium that causes disease such as fibrinous serositis, sometimes with caseous salpingitis and vegetative disorder [1]. *R. anatipestifer* infection is probably the most economically important disease of farmed ducks worldwide, but only a few virulence factors have been established so far, including VapD, CAMP cohemolysin and OmpA [2]–[5].

Lipopolysaccharide (LPS) is a major virulence factor of most Gram-negative bacteria [6]–[7]. The LPS molecule is typically composed of three parts: lipid-A, core-polysaccharide (core-PS) and O-antigen repeats. Lipid-A is the key constituent exhibiting endotoxic property which is anchored to the outer leaflet of the outer membrane. The core-PS is involved in immunomodulation, and is essential for the permeation properties of the bacterial outer membrane. The O-antigen repeats is known to be responsible for antigenicity and sero-specificity which is displayed on the surface of the bacterial cells [8]. There is an increasing body of evidence indicating that O-antigen plays an important role in its effective colonization of host tissue as well as in resistance to some bactericidal effects [9]–[10]. The genes for the synthesis of LPS have been characterized in several species of bacteria [11]–[12]. The biosynthesis of LPS is initiated in the cytoplasm and exported to the surface of bacteria. In *E. coli*, the biosynthetic pathway is well defined [13]–[14]. However, the genes responsible for *R. anatipestifer* LPS synthesis have not been characterized yet.

*R. anatipestifer* Yb2 is a serotype 2 virulent strain, which was isolated in Jiangsu province, China [15]. Infection with the Yb2 strain can kill 14–35 day-old domestic ducks within a mortality rate approaching 100% [16]. In this study, one LPS defected *R. anatipestifer* mutant strain RA2640 was identified by Tn4351 transposon mutagenesis, in which the AS87_04050 gene (GenBank accession no. KM455123) was inactivated. The mutant strain RA2640 showed a defected LPS molecule, changed LPS phenotype and sera-agglutination ability as well as attenuated bacterial virulence, indicating that the AS87_04050 gene is involved in *R. anatipestifer* LPS synthesis and responsible for *R. anatipestifer* virulence.

## Materials and Methods

### Bacterial strains, plasmids and growth conditions

The bacterial strains and plasmids used in this study are listed in Table 1. *R. anatipestifer* Yb2 is the wild-type strain used in this study, and the mutant strain RA2640 was derived from this strain by transposon insertion. The *Escherichia coli*–*Flavobacterium johnsoniae* shuttle plasmid pCP29 and *E. coli* strain BW19851, which carries the plasmid pEP4351, were obtained from Professor Mark J. McBride, University of Wisconsin-Milwaukee, USA. *R. anatipestifer* strains were grown on tryptic soy agar (TSA, Difco, USA) at 37°C for 24 h in 5% CO_2_ or tryptic soy broth (TSB, Difco). *E. coli* strains were grown at 37°C on Luria-Bertani (LB) plates or in LB broth. Antibiotics were used at the given concentrations when needed: ampicillin (100 µg/ml), chloramphenicol (30 µg/ml), erythromycin (0.5 µg/ml), kanamycin (50 µg/ml) and cefoxitin (5 µg/ml).

**Table 1:** Strains, plasmids and primers used in this study.

Strains, plasmids or primers	Characteristics	Source or reference
**Strains and plasmids**		
Yb2	*Riemerella anatipestifer* serotype 2 strain	This study
*Escherichia coli* S17–1	lpir hsdR pro thi; chromosomal integrated RP4–2 Tc::Mu Km::Tn7	17
RA2640	Tn4351 insertion mutant of *Riemerella. anatipestifer* Yb2, *AS87_04050*::Tn	This study
cRA2640	Mutant RA2640 carrying plasmid pCP29- AS87_04050	This study
pCP29	ColE1 ori; (pCP1 ori); Ap^r^(Em^r^); *E. coli*-*F. johnsoniae* shuttle plasmid	17
pCP- AS87_04050	pCP29 containing *ompA* promoter and AS87_04050 ORF, cfxA^r^ (Ap^r^)	This study
**Primers**		
RA 16S rRNA-F	5′-GGAGGCAGCAGTGAGGAATA-3′	This study
RA 16S rRNA-R	5′-ATCGTTTACGGCGTGGACTA-3′	This study
Erm-F	5′-GCCCGAAATGTTCAAGTTGT-3′	This study
Erm-R	5′-CTTGACAACCACCCGACTTT-3′	This study
Tn4351-F	5′ TGGCACCTTTGTGGTTCTTAC 3′	This study
Tn4351-R	5′ GAGAGACAATGTCCCCCTTTC 3′	This study
AS87_04040 F	5′-TCATGCAGTTTTGGAAGCAG-3′	This study
AS87_04040 R	5′-CGACGACCAGCCAAAATAAT-3′	This study
AS87_04050 F	5′-TTGGTAACGATTGGGAATCTC-3′	This study
AS87_04050 R	5′-TTACATCAGGCCGCTTTAGG-3′	This study
AS87_04055 F	5′-TATCTGGCTTAGGCGGAATG-3′	This study
AS87_04055 R	5′-GGTGAGGTTTCCCCGTAAAG-3′	This study
RA *ldh* F	5′-AACTTCCGCTTGGTATGCAC-3′	This study
RA *ldh* R	5′-TAGCCGCAGTAGCGAATTTT-3′	This study
ompA promoter P1	5′-CAGGTACCATAGCTAAAATTTTGGCAGTAAC-3′ (*Kpn* I site underlined)	17
ompA promoter P2	5′-CGACTCGAGCATTCCAATTCTCTTATTATC-3′ (*Xho* I site underlined)	17
AS87_04050 -comp-F	5′-CCGCTCGAGATGAATAACAGGAAAATATTAATTACTGGAGGGGCAGG-3′ (*Xho* I site underlined)	This study
AS87_04050 -comp-R	5′-CATGCATGCTTATTTCAACCTCTTCCAATACCAATCCAC-3′ (*Sph* I site underlined)	This study

### Identification of mutant strain RA2640

Tn4351 was introduced into *R. anatipestifer* wild-type strain Yb2 by conjugation from *E. coli* BW19851 as described previously [17]. The potential transconjugants appeared after 36–48 h of incubation at 37°C in 5% CO_2_ on selective medium. Primer pairs specific for *R. anatipestifer* strain (RA 16S rRNA-F/RA 16S rRNA-R) or Tn4351 (Erm-F/Erm-R) were described in Table 1 and used to identify mutants by PCR amplification. Mutants that failed to agglutinate *R. anatipestifer* serotype 2 positive sera (China Institute of Veterinary Drugs Control, Beijing, China) were selected for further analysis.

Southern blot analysis was used to identify the insertion of Tn4351 in the mutants. Briefly, genomic DNA of the mutant strain was purified using TIANamp Bacteria DNA kit (Tiangen, Beijing, China), digested with *Xba*I, separated by gel electrophoresis, and transferred to nylon membrane as previously described [17]. The *ermF* region of Tn4351 was amplified as a 626-bp PCR product from pEP4351 using primers Tn4351-F/Tn4351-R, and labeled with the DIG High Prime DNA Labeling and Detection Starter Kit I (Roche, Indianapolis, USA). Southern blot hybridization was performed by standard method according to the manufacturer's instructions. The plasmid pEP4351 and genomic DNA of wild-type strain Yb2 were also subjected to hybridization analysis, which were used as positive control and negative control respectively.

The site of transposon insertion in the mutant strain RA2640 was determined by inverse PCR [18]. Chromosomal DNA of the mutant was digested with *Hind*III and ligated, which resulted in the formation of closed circles. Tn4351-specific primers TN-1 and IS4351-F were used to amplify the DNA adjacent to the insertion site using *Premix LA Taq* (loading dye mix) (Takara, Dalian, China). DNA sequencing data were compared to database by using BLAST from National Center for Biotechnology Information (NCBI) website (http://www.ncbi.nlm.nih.gov/BLAST/).

Real-time RT-PCR was performed to measure levels of transcriptional expression of the genes upstream and downstream of the Tn4351-disrupted gene. Gene-specific primers were designed using primer3 online software v.0.4.0 (http://bioinfo.ut.ee/primer3-0.4.0/) and described in Table 1. The expression of the L-lactate dehydrogenase encoding gene (*ldh*) was measured using primers RA *ldh* F/RA *ldh* R (Table 1) and used as an internal control [19]. RNA was isolated using Trizol reagent (Invitrogen, Carlsbad, CA, USA), according to the manufacturer's instructions. Total RNA was treated with TURBO DNA-free kit (Ambion, Grand Island, NY, USA) to remove DNA contamination. cDNA was synthesized using PrimeScript RT Master Mix (Takara). Real-time RT-PCR was carried out in Go Taq qPCR Master Mix (Promega, Fitchburg, WI, USA) using the following parameters: 95°C for 2 min, 40 cycles of 95°C for 15 s, 55°C for 15 s and 68°C for 20 s, followed by one cycle of 95°C for 15 s, 60°C for 15 s and 95°C for 15 s. Reactions were performed in triplicate and run on the Mastercycler ep realplex4 apparatus (Eppendorf, Germany).

### Complementation of mutant strain RA2640

Complementation was accomplished by using the plasmid pCP29 [20]. The AS87_04050 gene was amplified from wild-type strain Yb2 using primers AS87_04050 comp-F/AS87_04050 comp-R (Table 1). The PCR product was inserted into pCP29 at *Xho*I and *Sph*I restriction sites, resulting in plasmid pCP29-AS87_04050. The expression of the AS87_04050 gene was under the control of the *ompA* promoter as described previously [17]. Plasmids were first introduced into *E. coli* S17–1 by transformation. Next, they were transferred into mutant strain RA2640 by conjugation. Transformants were selected on TSA containing 5 µg/ml cefoxitin and 50 µg/ml kanamycin, and identified by PCR amplification using primers AS87_04050 comp-F/AS87_04050 comp-R and RA 16S rRNA-F/RA 16S rRNA-R. The complementation strain was named as cRA2640.

### Bacterial growth curves

Wild-type strain Yb2, mutant strain RA2640 and complementation strain cRA2640 were grown in TSB respectively at 37°C for 8 h with shaking. The bacterial cultures were then transferred into fresh TSB medium at a ratio of 1∶50 (v/v) for further growing at 37°C for 16 h with shaking. The bacterial growth was measured as described [21], by monitoring the optical density at 600 nm (OD_600_) at 1 h intervals using a spectrophotometer (BIO-RAD, USA). The statistical significance of the data was determined by *t*-test in Graphpad Prism 5 software (GraphPad software, Inc., CA, USA). A *p* value of <0.05 was considered to be statistically significant.

### Autoaggregation assay

Autoaggregation assay was performed as described previously [22]. Briefly, the wild-type strain Yb2, mutant strain RA2640 and complementation strain cRA2640 were cultured to mid-logarithemic phase, and cooled in ice for 30 min to stop growing. The bacterial cultures were then shaken vigorously for 15 s and remained static at room temperature. At regular time intervals, a 100 µl sample was taken from the liquid surface and the OD_600_ values were measured using a spectrophotometer (BIO-RAD, USA).

### LPS extraction, purification and identification

LPS was extracted from the wild-type strain Yb2, mutant strain RA2640 and complementation strain cRA2640 using phenol-water method as described [23]. Briefly, bacterial cells were harvested by centrifugation at 10,000×g for 10 min, washed with 95% ethanol, acetone, and petroleum ether. After dried in the hood, the bacterial pellets were suspended in distilled water and subjected to LPS extraction. The LPS in the water phase was collected, washed twice with 95% ethanol and treated with proteinase K (150 µg/ml, Sangon, Shanghai, China), DNase I (10 µg/ml, Sangon) and RNase A (10 µg/ml, Sangon) to remove the contamination of protein and nucleic acids. After centrifugation at 1,000×g for 5 min, the supernatants were further ultracentrifuged (150,000×g) at 4°C for 3 h to pellet the LPS. The purified LPS was identified using SDS-PAGE followed by silver staining and commassie blue staining. Briefly, each LPS sample was dissolved in loading buffer at a concentration of 50 µg/ml and boiled at 100°C for 10 min. Samples were separated on 15% SDS gel at 100 V for 2 h. Gels were stained with silver to visualize the presence of LPS, and stained with commassie blue to exclude the contamination of protein.

### Bacterial resistance to antibiotics, disinfectants and normal duck sera

Bacterial resistance to ampicillin, gentamicin, spectinomycin, streptomycin sulfate, chloramphenicol, polymyxin B and nalidixic acid were performed and the minimal inhibitory concentration (MIC) was determined as described [15]. Briefly, each strain was adjusted to a standard turbidity of McFarland 0.5 and then applied to TSB containing continuous gradients of the antibiotics. The bacteria were statically incubated at 37°C for 24 h in 5% CO_2_, then the growth was monitored by measurement of the OD_600_. The MICs were determined as the lowest concentration at which there was no measurable growth.

Bacterial resistance to hydrogen peroxide and Triton X–100 was also determined. Hydrogen peroxide was diluted in PBS at final concentrations of 1.0 mM and 2.5 mM; Triton X–100 was diluted in PBS at final concentrations of 0.004% and 0.01% (v/v). To determine the viability of the bacteria exposed to disinfectants, 10 µl of cell suspension was inoculated into 190 µl 1.0 mM, 2.5 mM hydrogen peroxide or 0.004%, 0.01% Triton X–100 at a population of 2.5×10^6^ CFU. They were then incubated at 37°C for 30 min. After exposure to these disinfectants, bacteria were suitably diluted in PBS and plated on TSA plates. Then, the viability of the bacteria was determined.

Normal duck sera were collected from eight 21-day-old healthy Cherry Valley ducks, pooled and filter-sterilized (0.22 µm). The assay was performed as described [24]. Briefly, pooled sera were diluted to 12.5%, 25% and 50% in pH 7.2 PBS, or inactivated at 56°C for 30 min. Each 10 µl of bacterial suspension (2.5×10^8^ CFU/ml) was added into respective 190 µl of the diluted sera, the heat inactivated sera or PBS. The reaction mixtures were incubated at 37°C for 30 min, then 10-fold serial diluted and plated onto TSA plates. The plates were incubated at 37°C with 5% CO_2_ for 36 h for bacterial counting. The survival ratio was calculated by determining the ratio of colonies in the sera to those in the PBS.

### Adhesion and invasion assays

Adhesion and invasion assays were performed using Vero cells (ATCC CCL-81) as described [5]. Briefly, each well in a 24-well tissue culture plate was seeded with 10^5^ cells in Dulbecco's Modified Eagle Medium (DMEM) (Biowest, France) containing 10% fetal bovine serum. After incubation at 37°C with 5% CO_2_ for 20 h, the cell monolayer was rinsed with PBS and infected with bacterial cells at 50 or 100 multiplicity of infection (MOI), respectively. The plates were then incubated at 37°C with 5% CO_2_ for 1.5 h, washed three times with PBS and incubated at 37°C with 5% CO_2_ for 10 min in the presence of 0.1% trypsin (100 µl/well). The cell suspensions were 10-fold diluted and plated onto TSA plates to determine the number of viable bacterial cells. For the invasion assay, the extra-cellular bacteria were killed by incubation of the monolayer with DMEM medium supplemented with 100 µg/ml gentamicin for an additional 1 h, following the incubation with bacteria and three washes with PBS. All of the above assays were performed in triplicate and replicated three times.

### Determination of bacterial median lethal dose (LD_50_) and survival *in vivo*

The bacterial LD_50_ of the wild-type strain Yb2, mutant strain RA2640 and complementation strain cRA2640 were determined respectively as described [5]. Briefly, the mid-logarithemic bacterial cultures were suspended in 0.5 ml PBS at 10^4^–10^11^ colony forming units (CFU). One-day-old Cherry Valley ducks were purchased from Zhuanghang duck farm (Fengxian District, Shanghai). A total of 104 ducks were housed in cages with a 12-h light/dark cycle and free access to food and water during the study. The ducks were divided randomly at 21-days-old into 13 groups (8 ducks per group) and injected intramuscularly with respective bacterial dilutions (10^4^ to 10^11^ CFU) in 0.5 ml PBS. Ducks in groups 1–4 were injected with 10^4^, 10^5^, 10^6^ or 10^7^ CFU of Yb2 bacteria; Ducks in groups 5–9 were injected with 10^7^, 10^8^, 10^9^, 10^10^ or 10^11^ CFU of RA2640 bacteria; Ducks in groups 10–13 were injected with 10^4^, 10^5^, 10^6^ or 10^7^ CFU of cRA2640 bacteria. Ducks that became moribund (clinical signs include diarrhea, rough hair coat, frequent seizure activity, paralysis and no eating or drinking, etc.) were euthanized humanely with an intravenous injection of sodium pentobarbital at a dose of 120 mg/kg and counted as dead. Dead ducks were subjected to *R. anatipestifer* identification. Ducks was monitored daily for clinical symptoms and death rate until 7 days post-infection. The LD_50_ was calculated by improved Karber's method [25].

21-day-old ducks were inoculated intramuscularly with the wild-type strain Yb2, mutant strain RA2640 and complementation strain cRA2640 at 10^7^ CFU in 0.5 ml PBS to determine the bacterial survival *in vivo* [16]. Blood samples were collected at 3, 6, 12 and 24 hpi, 10-fold diluted and plated in triplicate on TSA. Plates were incubated at 37°C with 5% CO_2_ for 36 h for bacterial counting. Survival rate *in vivo* was determined for each mutant by comparison to the wild-type strain controls.

### Ethics Statement

This study was carried out in strict accordance with the recommendations in the Guide for the Care and Use of Laboratory Animals of the Institutional Animal Care and Use Committee (IACUC) guidelines set by Shanghai Veterinary Research Institute, the Chinese Academy of Agricultural Sciences (CAAS). The protocol was approved by the Committee on the Ethics of Animal Experiments of Shanghai Veterinary Research Institute, CAAS (Permit Number: 13-08). All surgery was performed under sodium pentobarbital anesthesia, and all efforts were made to minimize suffering.

## Results

### Characterization of Tn4351-induced mutant strain RA2640

**Figure 1:**
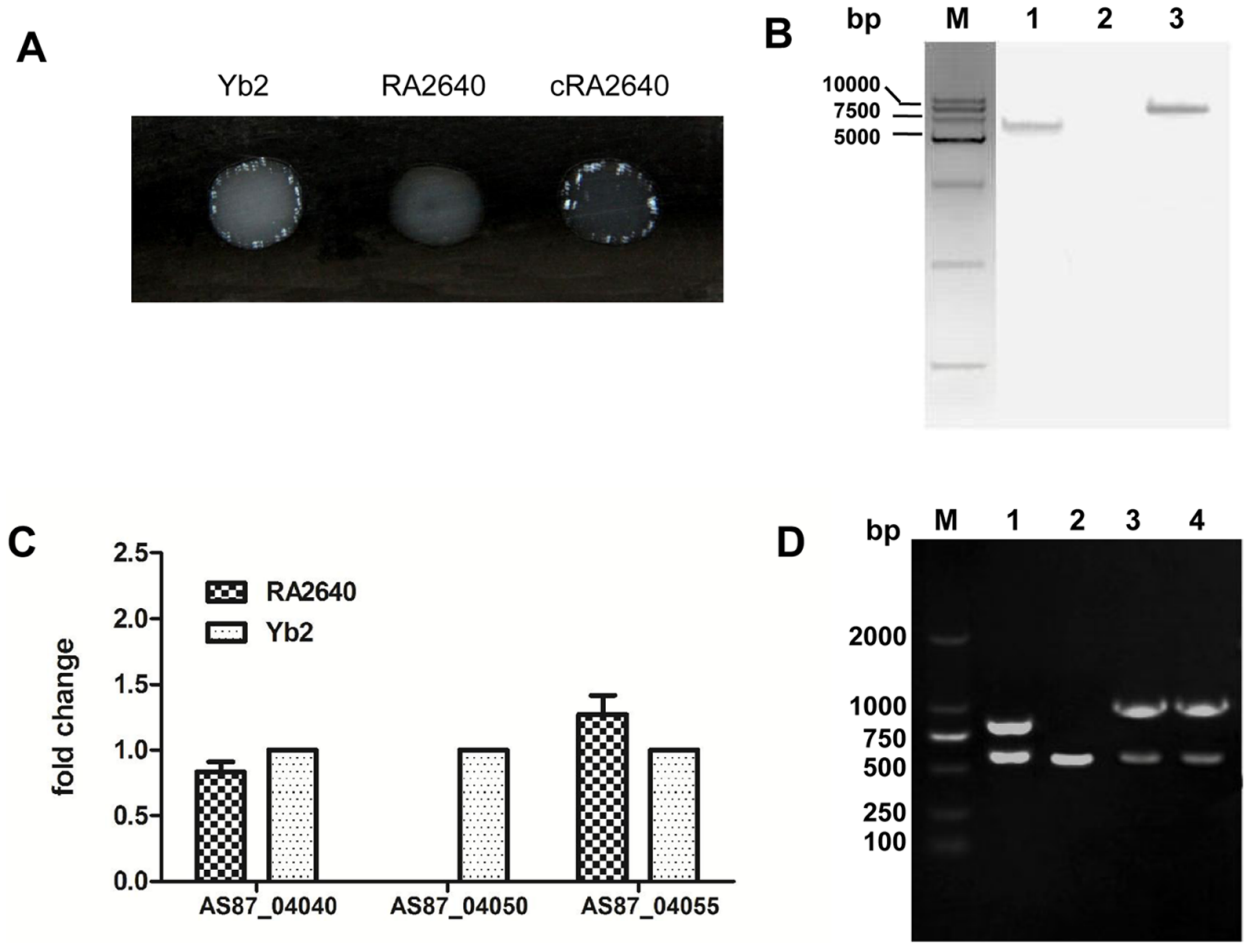
Construction and characterization of *R. anatipestifer* mutant strain RA2640 and complementation strain cRA2640. (A) Slide agglutination test: Mutant strain RA2640 lacked serum agglutination ability to serotype 2 positive sera. (B) Southern blot analysis. Lane M: DM15000 DNA Marker (CWBIO, Beijing, China). Lane 1: A single insertion of Tn4351-transposon was identified in mutant strain RA2640. Lane 2: No hybridization band was detected in wild-type strain Yb2. Lane 3: *Xba*I fragment (10.4 kb) of pEP4351 was identified with the *ermF* gene probe. (C) Real-time RT-PCR analysis. The expression of upstream AS87_04040 gene and downstream AS87_04055 gene were measured. The changes of mRNAs were expressed as fold expression and calculated using the comparative C_T_ (2^-△△CT^) method. The expression of AS87_04050 in mutant strain RA2640 couldn't be detected. Error bars represent SD from three replicates. (D) PCR analysis. Lane M: DM2000 DNA Marker (CWBIO, Beijing, China). Lane 1: The Erm and *R. anatipestifer* 16S rRNA were amplified from the mutant strain RA2640 using primer pairs Erm-F/Erm-R and RA 16S rRNA-F/RA 16S rRNA-R, showing an 833-bp fragment of Erm or a 496-bp fragment of RA 16S rRNA. Lane 2: A 496-bp fragment was amplified from wild-type strain Yb2 with primers RA 16S rRNA-F/RA 16S rRNA-R. Lane 3: The AS87_04050 gene and *R. anatipestifer* 16S rRNA were amplified from the complementation strain cRA2640 using primer pairs AS87_04050 comp-F/AS87_04050 comp-R and RA 16S rRNA-F/RA 16S rRNA-R, showing a 972-bp fragment of AS87_04050 or a 496-bp fragment of RA 16S rRNA.. Lane 4: The AS87_04050 gene and *R. anatipestifer* 16S rRNA were amplified from wild-type strain Yb2 using primer pairs AS87_04050 comp-F/AS87_04050 comp-R and RA 16S rRNA-F/RA 16S rRNA-R, showing a 972-bp fragment of AS87_04050 or a 496-bp fragment of RA 16S rRNA.

In the initial screening of *R. anatipestifer* Yb2 mutants carrying transposon Tn4351, we identified one mutant strain RA2640 by a slide agglutination test, which lacked serum agglutination ability to serotype 2 positive sera (Fig. 1A). Insertion of Tn4351 into the RA2640 chromosome was confirmed by Southern blot, the hybridized band revealed that RA2640 had a single Tn4351 insertion (Fig. 1B). The DNA sequence surrounding the transposon insertion was obtained by inverse PCR. Sequencing of the cloned DNA fragment revealed that the insertion site was located at 288 bp of AS87_04050 gene in wild-type strain Yb2. The AS87_04050 gene is 972 nucleotides in length and codes for a predicted nucleoside-diphosphate-sugar epimerase consisting of 323 amino acids. BLAST analysis showed that AS87_04050 gene was highly conserved in *R. anatipestifer*, which exhibits 99%, 99%, 96% and 95% identity compared to RA-CH-2 (GenBank accession no. CP004020.1), RA-GD (GenBank accession no. CP002562.1), RA-CH-1 (GenBank accession no. CP003787.1) and DSM15868 (GenBank accession no. CP002346.1), respectively. In addition, the predicted protein exhibits sequence identity to predicted UDP-glucose 4-epimerase (GalE) protein from other bacteria such as *Weeksella virosa* (GenBank accession no. YP_004238251.1; 80%), *Flavobacteriaceae bacterium* (GenBank accession no. YP_003094858.1; 78%) and *Cellulophaga geojensis* (GenBank accession no. EWH14022.1; 73%). The predicted protein of AS87_04050 also exhibits sequence similarity to polysaccharide biosynthesis protein from other micro-organisms, including *Flavobacterium limnosediminis* (GenBank accession no. WP_023579572.1; 75%), *Gramella forsetii* KT0803 (GenBank accession no. YP_862073.1; 69%) and *Treponema primitia* ZAS-2 (GenBank accession no. YP_004523536.1; 68%).

The expression of the upstream AS87_04040 gene, which encodes UDP-N-acetyl-D-mannosaminuronate dehydrogenase, and the downstream AS87_04055 gene, which encodes uncharacterized protein involved in exopolysaccharide biosynthesis were investigated by qRT-PCR using the L-lactate dehydrogenase gene (*ldh*) for normalization (data not shown). As shown in Fig. 1C, the expression of both AS87_04040 and AS87_04055 genes in mutant strain RA2640 showed no significant changes, compared with wild-type strain Yb2. No expression of Tn4351-disrupted AS87_04050 gene was detected in the mutant strain RA2640 by real-time RT-PCR.

Plasmid pCP29-AS87_04050, which carries an AS87_04050 gene under the control of the *R. anatipestifer ompA* promoter, was constructed and used for complementation of mutant strain RA2640. The complementation strain was identified by PCR amplification (Fig. 1D) and designated as cRA2640. Slide agglutination test showed that cRA2640 recovered the ability to agglutinate serotype 2 positive serum. The results of the complementation studies are in agreement with real-time RT-PCR results, indicating that the AS87_04050 gene is responsible for above changed characteristics of mutant strain RA2640.

### Bacterial growth and phenotype identification

**Figure 2:**
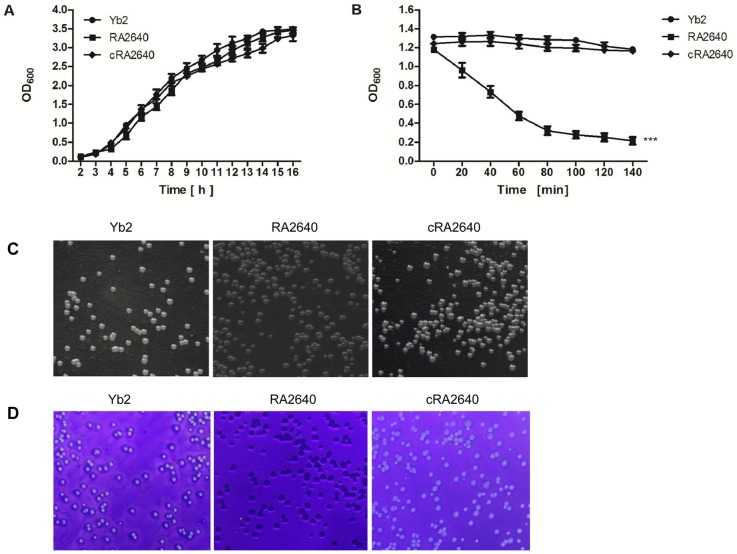
Characterization of the bacterial growth and phenotype. (A) Bacterial growth curves: No significant difference was shown among *R. anatipestifer* wild-type strain Yb2, mutant strain RA2640 and complementation strain cRA2640. (B) Autoaggregation assay: Mutant strain RA2640 was shown in a settling pattern from static liquid suspensions. The data represent the mean ± standard error of three separate experiments with each strain in duplicate. ***, *p*<0.001. (C) Colony morphology: The wild-type strain Yb2 and complementation strain cRA2640 on TSA was smooth, slightly raised and transparent, whereas mutant strain RA2640 was rough and non-transparent. (D) Crystal violet staining: Mutant strain RA2640 was stained by crystal violet.

The bacterial growth in TSB was examined, no significant differences were found among *R. anatipestifer* wild-type strain Yb2, mutant strain RA2640 and complementation strain cRA2640 (Fig. 2A, *p*>0.05). Autoaggregation assay showed that *R. anatipestifer* wild-type strain Yb2 did not aggregate or settle from liquid suspensions, however, the mutant strain RA2640 was observed to flocculate and settle from standing overnight culture. The efficiency of autoaggregation was significantly increased in the mutant RA2640 (Fig. 2B, *p*<0.001). The complementation strain cRA2640 recovered the non-autoaggregation character of the wild-type strain Yb2, indicating that inactivation of AS87_04050 gene increased the bacterial autoaggregation. The colony morphology of wild-type strain Yb2 on TSA was smooth, slightly raised and transparent, whereas mutant strain RA2640 was rough and non-transparent (Fig. 2C). The crystal violet staining assay was performed as described [26], showed that the mutant strain RA2640 was stained by crystal violet, while the wild-type strain Yb2 and complementation strain cRA2640 couldn't be stained (Fig. 2D). Combined with the results of autoaggregation assay, we conclude that inactivation of AS87_04050 gene changed the smooth-phenotype of wild-type strain Yb2 to rough-phenotype of mutant strain RA2640.

### Analysis of the bacterial LPS by SDS-PAGE and silver staining

**Figure 3:**
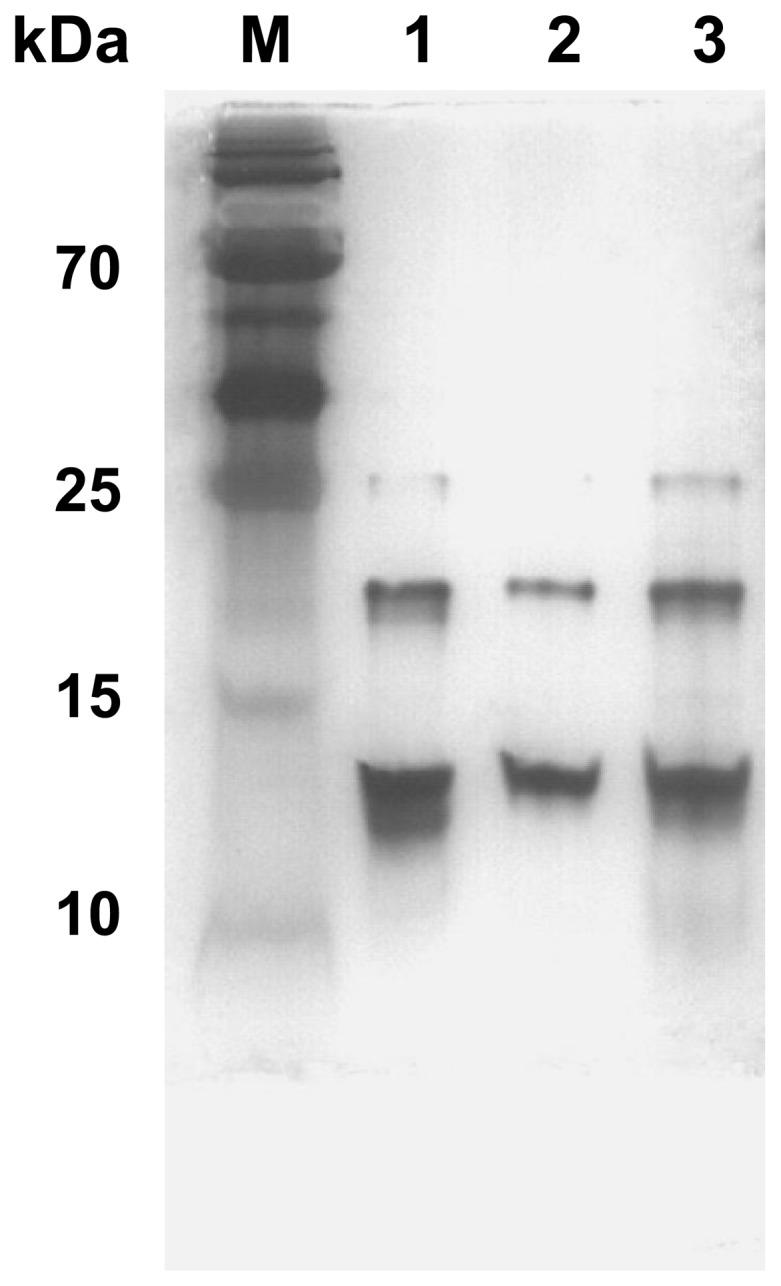
Silver staining of purified LPS. LPS was purified by hot phenol-water method, fractionated over SDS-PAGE, and stained by silver. Each lane contains 0.5 µg of LPS. Lane 1: Wide-type strain Yb2 LPS. Lane 2: Mutant strain RA2640 LPS. Lane 3: Complementation strain cRA2640 LPS.

To determine whether the LPS of mutant strain RA2640 was defected, the LPS was purified from the wild-type strain Yb2, mutant strain RA2640 and complementation strain cRA2640 and subjected to SDS-PAGE and silver staining. As shown in Fig. 3, LPS obtained from the wild-type strain Yb2 displayed three bands at 13 kDa, 20 kDa and 25 kDa, while the LPS of mutant strain RA2640 displayed two bands at 13 kDa and 20 kDa, the 25 kDa band was absent. The complementation strain cRA2640 recovered three bands of wild-type strain Yb2 LPS. Coomassie blue staining showed no detectable protein bands in the gels (data not shown). These results are beginning to suggest that *R. anatipestifer* AS87_04050 gene may play some roles in LPS synthesis.

### Mutant strain RA2640 displayed a higher sensitivity to antibiotics, disinfectants and normal duck sera

**Figure 4:**
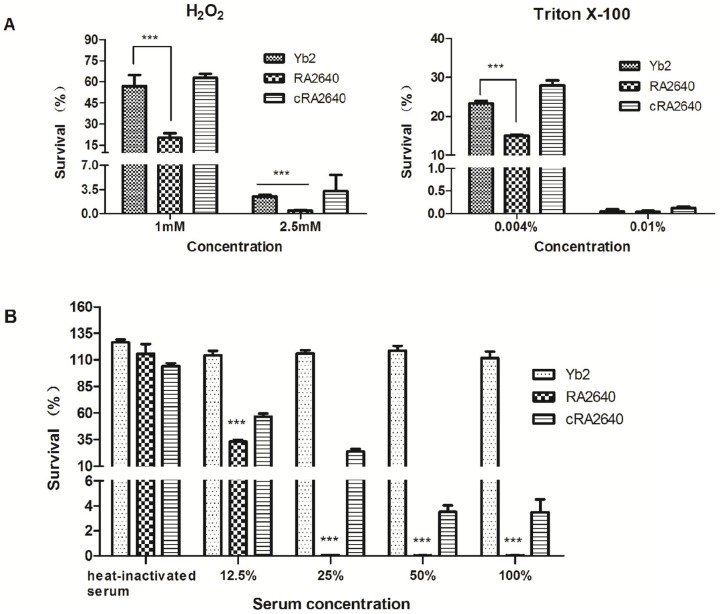
Sensitivity to disinfectants and normal duck sera. (A) Susceptibilities to hydrogen peroxide and Triton X–100, expressed as survival rate after incubation of bacteria with disinfectants at 37°C for 30 min as compared to PBS. Bars represent the means ± standard deviation of the results found in three experiments. (B) Susceptibilities to normal duck sera, expressed as survival rate after incubation of bacteria with normal duck sera at 37°C for 30 min at different dilutions as compared to PBS. ***, *p*<0.001. Error bars represent the standard deviation of three independent experiments.

The microdilution assay showed that the MICs of gentamicin, chloramphenicol and ampicillin for the mutant strain RA2640 were 2–4 times lower than those of wild-type strain Yb2, suggesting that RA2640 was more susceptible to antibiotic (Table 2). In addition, the susceptibility assay showed that mutant strain RA2640 was more sensitive to the treatment of hydrogen peroxide or Triton X–100 than wild-type strain Yb2 presented (Fig. 4A). After 30 min exposure to 1.0 mM or 2.5 mM hydrogen peroxide, the survival rates of mutant strain RA2640 were 20.15% and 0.43% respectively, which were significantly lower than those of wild-type strain Yb2 (56.93% and 2.48% respectively, *p*<0.001). At the end of 30 min exposure period to 0.01% Triton X–100, all test strains exhibited lower than 1% survival. Meanwhile, a significantly lower (*p*<0.001) survival of 14.99% was noted with the mutant strain RA2640 exposed to 0.004% Triton X–100 when compared with the wild-type strain Yb2 (23.08%). The complementation strain cRA2640 recovered the resistance of Yb2 to antibiotics and disinfectants.

**Table 2:** Bacterial MICs determination.

Antibiotics^a^	MIC (µg/ml)		
	Yb2	RA2640	cRA2640
Ampicillin	1	0.25	8
Streptomycin sulfate	10	10	25
Gentamicin	25	12.5	50
Spectinomycin sulfate	75	50	50
Chloramphenicol	12.5	6.25	4
Polymyxin B	120	100	100
Nalidixic acid	135	125	135

a The concentrations tested for ampicillin were 0.125, 0.25, 0.5, 1.0, 2.0, 4.0, 8.0 and 10 µg/ml respectively; for streptomycin sulfate were 5, 10, 15, 20, 25 and 30 µg/ml respectively; for gentamicin were 6.25, 12.5, 25, 50 and 60 µg/ml respectively; for spectinomycin sulfate were 25, 50, 75 and 100 µg/ml respectively; for chloramphenicol were 4, 6.25, 12.5 and 25 µg/ml respectively; for polymyxin B were 80, 100, 120 and 140 µg/ml respectively; and for nalidixic acid were 115, 125, 135 and 145 µg/ml, respectively.

To determine whether AS87_04050 gene is involved in serum resistance of the wild-type strain Yb2, a serum bactericidal assay was carried out. The results showed that the survival rates of mutant strain RA2640 were 33.10%, 0.03%, 0.02% and 0.02% in 12.5%, 25%, 50% and 100% duck serum, respectively. In contrast, wild-type strain Yb2 was serum resistant, demonstrating 114.27%, 115.92%, 118.70% and 111.72% survival rates in 12.5%, 25%, 50% and 100% duck serum, respectively. The survival rates of the mutant strain RA2640 in normal duck sera were clearly reduced compared to the wild-type strain Yb2 strain (*p*<0.001) (Fig. 4B). Complementation strain cRA2640 partly restored the serum-resistant ability, exhibiting 56.55%, 23.98%, 3.53% and 3.48% survival rates in 12.5%, 25%, 50% and 100% duck serum, respectively. Thus, *R. anatipestifer* AS87_04050 gene is associated with the bacterial resistance to normal sera.

### Inactivation of AS87_04050 gene increased the bacterial adherence and invasion abilities

**Figure 5:**
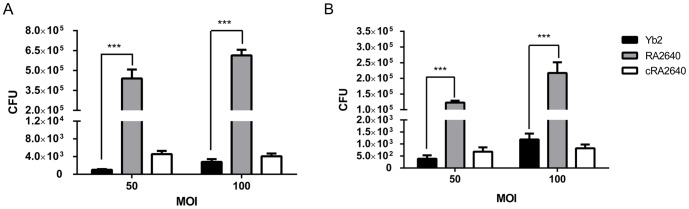
Bacterial adherence and invasion assays. The assays were performed in Vero cells. (A) Adherence assay. (B) Invasion assay. Vero cells cultured in 24-well plates were infected with wild-type strain Yb2, mutant strain RA2640 or complementation strain cRA2640 at 50 or 100 MOI and incubated for 1.5 h to count adhered bacterial CFU. For the invasion assay, the extra-cellular bacteria were killed by incubation of the monolayer in DMEM with 100 µg/ml gentamicin for an additional 1 h. The data represent the number of bacteria that adhered to or invaded into the cells in each well of 24-well plates. Error bars represent the standard deviation from three independent experiments performed in triplicate. ***, *p*<0.001.

Vero cells were utilized to compare the bacterial adherence and invasion abilities. The adhered RA2640 bacteria were counted as 4.4×10^5^ CFU/well or 6.14×10^5^ CFU/well respectively, when infected at 50 or 100 multiplicity of infection (MOI), which was 428 or 218 times higher than that of wild-type strain Yb2 (1,026 CFU/well at an MOI of 50; 2,806 CFU/well at an MOI of 100) (*p*<0.001). After an additional 1 h of incubation with gentamicin (100 µg/ml), the bacterial counts for mutant strain RA2640 were 1.23×10^5^ CFU/well at 50 MOI and 2.17×10^5^ CFU/well at 100 MOI, which were significantly increased in comparison with those of wild-type strain Yb2 (*p*<0.001). The results showed that adhesion and invasion capacities of mutant strain RA2640 were significantly increased compared to that of wild-type strain Yb2 (*p*<0.001), and complementation strain cRA2640 restored the adhesion and invasion capacities (Fig. 5).

### Determination of the bacterial virulence

In order to elucidate the effect of mutation on the bacterial virulence, LD_50_ of the wild-type strain Yb2, mutant strain RA2640 and complementation strain cRA2640 were measured using Cherry Valley ducks. The results showed that the LD_50_ for Yb2, RA2640 and cRA2640 were 1.07×10^5^, 1.91×10^10^ and 7.08×10^5^ CFU respectively (Table 3). The virulence of mutant strain RA2640 was approximately 100,000-fold attenuated, compared to the wild-type strain Yb2. The complementation strain cRA2640 restored the bacterial virulence of wild-type strain Yb2. The result indicated that AS87_04050 gene is required for full virulence of *R. anatipestifer* strain Yb2.

**Table 3:** Bacterial LD_50_ determination.

Strains	LD_50_ (CFU)
Yb2	1.07×10^5^
RA2640	1.91×10^10^
cRA2640	7.08×10^5^

**Figure 6:**
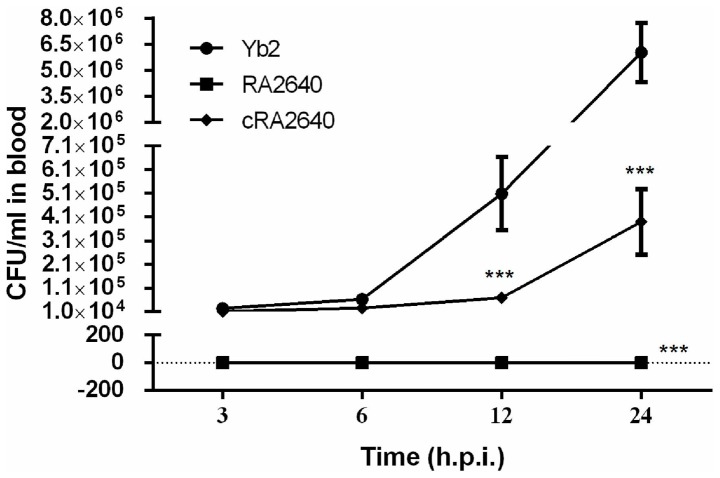
Bacterial loads in blood of *R. anatipestifer* infected ducks. Six ducks were injected intramuscularly with bacterial culture at 1×10^7^ CFU. Blood samples were collected at 3, 6, 12 and 24 h.p.i. and serially diluted for bacterial counting. Each point represents the mean ± standard deviation.

To assess systemic invasion and dissemination, the kinetics of wild-type strain Yb2, mutant strain RA2640 and complementation strain cRA2640 were determined after bacterial inoculation by quantifying the CFU present in the blood at different time points. Results showed that wild-type strain Yb2 was recovered from blood at a quantity of 2.51×10^4^ CFU/ml at 3 h.p.i, and 6.04×10^6^ CFU/ml at 24 h.p.i. (Fig. 6). However, no bacteria were recovered from blood of ducks infected with mutant strain RA2640 at all time points detected. Complementation strain cRA2640 recovered the bacterial loads of 1.14×10^4^ and 2.57×10^4^ CFU/ml in the blood at 3 and 6 h.p.i.. At 12 and 24 h.p.i., the bacterial loads were partly recovered to 6.95×10^4^ and 3.89×10^5^ CFU/ml respectively (Fig. 6). These results indicated that mutant strain RA2640 could not survive in the duck blood after infection, even as early as 3 h.p.i.

## Discussion

DNA transposition, in which the transposon acts as an insertion mutagen, is a powerful approach for the generation of appropriate knockout mutations for functional gene analysis. Our previous study has identified 33 genes which were involved in *R. anatipestifer* biofilm formation by random transponson mutagenesis [17]. In the present study, we identified one mutant RA2640, which had defects in serum agglutination ability, LPS biosynthesis, and resistance to antibiotics, disinfectants and normal duck serum. Animal experiments further demonstrated that the virulence of mutant strain RA2640 was reduced significantly, compared to its wild-type strain Yb2. Sequence analysis revealed that the transposon was inserted into the AS87_04050 gene at 288 bp in strain Yb2.

The AS87_04050 gene encodes a putative nucleoside-diphosphate-sugar epimerase, which belongs to the short chain dehydrogenase/reductase superfamily [27]. The proteins in this family utilize NAD as a cofactor and use nucleotide-sugar substrates for a variety of chemical reactions. In *Vibrio vulnificus*, the epimerase is involved in the synthesis of extracellular polysaccharide capsule components, as well as metabolic pathways [28]. AS87_04050 gene shared 95–99% identity with *R. anatipestifer* strains DSM15868, RA-CH-1, RA-GD and RA-CH-2. To investigate whether the AS87_04050 gene is involved in the LPS synthesis of *R. anatipestifer* strain Yb2, LPS was isolated from wild-type strain Yb2, mutant strain RA2640 and complementation strain cRA2640, and analyzed by SDS-PAGE followed by silver staining. LPS isolated from mutant strain RA2640 showed a truncated LPS profile, indicating that inactivation of the AS87_04050 gene resulted in the defection of the LPS integrity, and the AS87_04050 gene is associated with the biosynthesis of intact LPS in *R. anatipestifer*. Mutant strain RA2640 was shown to autoaggregate and was stained by crystal violet, suggesting that the phenotype of the mutant RA2640 was changed to rough style from its smooth style of wild-type strain Yb2. These data are additional evidences that the AS87_04050 gene is responsible for the LPS synthesis. Defects in LPS biosynthesis in the mutant strain RA2640 led to changes in cell surface structure, which may be the reason for the observation of flocculated and settled bacteria from standing overnight cultures. In addition, the mutant strain RA2640 was found to exhibit the acervate phenotype, in contrast to the wild-type strain Yb2. This phenotype may be due to a failure of cellular segregation resulting from the cell surface.

Mutant strain RA2640 lost the ability to agglutinate serotype 2 positive sera, which is the additional evidence that the AS87_04050 gene is responsible for O-antigen synthesis in *R. anatipestifer*. LPS is the major component of the outer membrane of Gram-negative bacteria, contributing greatly to the structural integrity of the bacteria, and protecting the membrane from certain kinds of chemical attack. Our results showed that inactivation of AS87_04050 gene in mutant strain RA2640 raised the bacterial sensitivity to antibiotic and disinfectant, which are consistant with the previous reports [29].

The ability of bacteria to produce systemic infection often corresponds to resistance to the bactericidal activity of the host complement, which allows bacteria effectively to evade immune responses and to survive in the blood stream [30]. Thus, serum resistance represents an important virulence strategy of bacterial pathogens. Moreover, previous studies have indicated that LPS is required for serum resistance [31]–[32]. In *Burkholderia pseudomallei* serum-sensitive mutant, the type II O-antigenic polysaccharide moiety of LPS was not present [33]. In *Salmonella*, LPS O-antigen chain length is an important factor that protects bacteria from serum complement [34]. In this study, inactivation of the AS87_04050 gene resulted in significantly increased sensitivity to normal duck serum killing, with the extremely low level survival of mutant strain RA2640 in normal duck sera.

It is well established that LPS is a major factor in the virulence of Gram-negative bacteria. The contribution of LPS to pathogenesis and immunity varies depending on the underlying animal basis for increased susceptibility to infection, the isoform of the LPS, particularly the lipid A component, and structural variation in the O-antigen side chain that impacts host immunity. For example, *Brucella* possesses an unconventional non-endotoxic LPS that confers resistance to anti-microbial attacks and modulates the host immune response [6]. In *Haemophilus influenza*, host-derived sialic acid is incorporated into LPS and sialylated LPS glycoforms play a key role in pathogenesis of experimental otitis media [35]. The LPS of *Campylobacter jejuni* is identified as a potential adhesion [36], which specifically bound to epithelial cells, and this phenomenon was inhibited by periodate oxidation of the LPS or glutaradehyde fixation of the epithelial cells [37]. In an effort to explore the role of O-antigen in the interaction between *R. anatipestifer* and epithelial cells, we have investigated the adherence and invasion capability of mutant strain RA2640 by using the Vero cells. The results confirmed a more than 100-fold increased numbers of adhesion and invasion of mutant strain RA2640 than that of wild-type strain Yb2. That is to say, rough mutant exhibited increased epithelial cells uptake relative to its smooth wild-type strain, which is also observed in other bacteria [38]. For instance, *B. abortus* rough mutants are taken up in greater numbers by macrophages than the smooth parental strains and this increased uptake and replication coincide with necrotic cell death of the macrophages [39], [40]. Likewise, the increased adherence and invasion capability to Vero cells for the mutant strain RA2640 might be dependent on the rough phenotype of LPS, since the bacteria with rough phenotype may take different way to adherent and invade cells as reported for *B. abortus* [40].

To explore the role of the AS87_04050 gene in the bacterial pathogenicity, we have infected the 21-day old Cherry Valley ducks with wild-type strain Yb2, mutant strain RA2640 and complementation strain cRA2640. As a result, mutant strain RA2640 were not recovered from infected animals as Yb2 did, and showed significant reductions in its ability to colonize ducks, compared to the wild-type strain Yb2. LD_50_ measurement further indicated the virulence of mutant strain RA2640 was more than 100,000 times attenuated than that of wild-type strain Yb2. Therefore, animal experiments confirmed that intact LPS is important for *R. anatipestifer* to establish a systemic infection.

In summary, we have identified one mutant strain RA2640, in which the LPS biosynthesis was defected due to the inactivation of AS87_04050 gene. Moreover, the mutant strain RA2640 was unable to cause systemic infection and the virulence was significantly attenuated. We conclude that the AS87_04050 gene in *R. anatipestifer* is associated with the LPS biosynthesis and bacterial pathogenicity.
